# Increased Intracellular Cyclic di-AMP Levels Sensitize Streptococcus gallolyticus subsp. gallolyticus to Osmotic Stress and Reduce Biofilm Formation and Adherence on Intestinal Cells

**DOI:** 10.1128/JB.00597-18

**Published:** 2019-02-25

**Authors:** Wooi Keong Teh, Shaynoor Dramsi, Tim Tolker-Nielsen, Liang Yang, Michael Givskov

**Affiliations:** a Singapore Centre for Environmental Life Sciences Engineering, Nanyang Technological University, Singapore, Singapore; b Interdisciplinary Graduate School, Nanyang Technological University, Singapore, Singapore; c Institut Pasteur, Unité de Biologie des Bactéries Pathogènes à Gram-Positif, Paris, France; d Costerton Biofilm Center, Department of Immunology and Microbiology, University of Copenhagen, Copenhagen, Denmark; e School of Biological Sciences, Nanyang Technological University, Singapore, Singapore; f Centre National de la Recherche Scientifique (CNRS) ERL6002, Paris, France; NCBI, NLM, National Institutes of Health

**Keywords:** *Streptococcus gallolyticus*, c-di-AMP, *Streptococcus bovis*, biofilm, cell adherence

## Abstract

Cyclic di-AMP is a recently identified second messenger exploited by a number of Gram-positive bacteria to regulate important biological processes. Here, we studied the phenotypic alterations induced by the increased intracellular c-di-AMP levels in Streptococcus gallolyticus, an opportunistic pathogen responsible for septicemia and endocarditis in the elderly. We report that an S. gallolyticus c-di-AMP phosphodiesterase *gdpP* knockout mutant, which displays a 1.5-fold higher intracellular c-di-AMP levels than the parental strain UCN34, is more sensitive to osmotic stress and is morphologically smaller than the parental strain. Unexpectedly, we found that a higher level of c-di-AMP reduced biofilm formation of S. gallolyticus on abiotic surfaces and reduced adherence and cell aggregation on human intestinal cells. A genome-wide transcriptomic analysis indicated that c-di-AMP regulates many biological processes in S. gallolyticus, including the expression of various ABC transporters and disease-associated genes encoding bacteriocin and Pil3 pilus. Complementation of the *gdpP* in-frame deletion mutant with a plasmid carrying *gdpP* in *trans* from its native promoter restored bacterial morphology, tolerance to osmotic stress, biofilm formation, adherence to intestinal cells, bacteriocin production, and Pil3 pilus expression. Our results indicate that c-di-AMP is a pleiotropic signaling molecule in S. gallolyticus that may be important for S. gallolyticus pathogenesis.

**IMPORTANCE** Streptococcus gallolyticus is an opportunistic pathogen responsible for septicemia and endocarditis in the elderly and is also strongly associated with colorectal cancer. S. gallolyticus can form biofilms, express specific pili to colonize the host tissues, and produce a specific bacteriocin allowing killing of commensal bacteria in the murine colon. Nevertheless, how the expression of these colonization factors is regulated remains largely unknown. Here, we show that c-di-AMP plays pleiotropic roles in S. gallolyticus, controlling the tolerance to osmotic stress, cell size, biofilm formation on abiotic surfaces, adherence and cell aggregation on human intestinal cells, expression of Pil3 pilus, and production of bacteriocin. This study indicates that c-di-AMP may constitute a key regulatory molecule for S. gallolyticus host colonization and pathogenesis.

## Abstract (precis)

Streptococcus gallolyticus is an opportunistic pathogen responsible for septicemia and endocarditis in the elderly and is also strongly associated with colorectal cancer. S. gallolyticus can form biofilms, express specific pili to colonize the host tissues, and produce a specific bacteriocin allowing killing of commensal bacteria in the murine colon. Nevertheless, how the expression of these colonization factors is regulated remains largely unknown. Here, we show that c-di-AMP plays pleiotropic roles in S. gallolyticus, controlling the tolerance to osmotic stress, cell size, biofilm formation on abiotic surfaces, adherence and cell aggregation on human intestinal cells, expression of Pil3 pilus, and production of bacteriocin. This study indicates that c-di-AMP may constitute a key regulatory molecule for S. gallolyticus host colonization and pathogenesis.

## INTRODUCTION

Streptococcus gallolyticus subsp. gallolyticus was previously known as Streptococcus bovis biotype I. This Gram-positive bacterium belonging to the group D *Streptococcus* is an emerging pathogen responsible for septicemia and infective endocarditis (IE) in the elderly. S. gallolyticus infections represent up to 25% of all cases of IE (1) and up to 94.5% of IE caused by group D *Streptococcus* (2). Once established, IE is usually difficult to treat and has an in-hospital mortality of up to 22% (3–5), and high-risk surgery interventions are often needed to resolve the infection (4–6). Importantly, S. gallolyticus also has a strong association with the occurrence of colorectal cancer in endocarditis patients (7, 8). Two recent studies indicated that S. gallolyticus is both a driver and a passenger in colonic tumorigenesis. On one hand, S. gallolyticus subsp. gallolyticus can accelerate colorectal cancer development by inducing cell proliferation through the β-catenin pathway (9); on the other hand, S. gallolyticus can take advantage of tumoral conditions to outcompete the colonic microbiota members, such as Enterococcus faecalis, through the production of a specific bacteriocin named gallocin (10). S. gallolyticus was also shown to express two specific pili named Pil1 and Pil3, which are involved in collagen and mucin binding, respectively. These two specific pili allow S. gallolyticus to attach at infection sites (i.e., cardiac valves and colon, respectively) and play a role in biofilm formation (11, 12). While these studies provided important clues to understand S. gallolyticus pathogenicity, not much is known about the signal(s) governing the induction of virulence.

In bacteria, second messenger signaling molecules are exploited to regulate important physiological functions, including biofilm formation and virulence. Among these molecules, cyclic di-AMP (c-di-AMP) has gained attention due to its widespread presence in Gram-positive bacteria, its essentiality for survival in certain conditions, and its pleiotropic role as both an intracellular and extracellular molecule modulating many biological processes, including the host immune responses (13–16).

c-di-AMP is synthesized by DisA_N or DAC domain-containing diadenylate cyclases (DACs) and hydrolyzed by DHH/DHHA1 or HD-domain-containing phosphodiesterases (PDEs) (17–21). Unlike *Bacillus* and *Clostridium* spp., which carry multiple DACs in their genomes, *Firmicutes*, such as *Streptococcus* and *Staphylococcus* spp., are known to carry only one DAC (commonly encoded by *dacA*) (15, 18, 22). Previous attempts to delete *dacA* from Listeria monocytogenes and Streptococcus pyogenes and all DACs from Bacillus subtilis using conventional gene deletion protocols were unsuccessful (13, 22–24), suggesting that c-di-AMP is essential for bacterial growth in standard laboratory culturing conditions. It is now known that c-di-AMP is dispensable for growth in specially formulated media and under anaerobic culturing conditions (25–28). However, the deletion of *dacA* often results in the occurrence of compensatory mutations (27, 28). Therefore, c-di-AMP PDE deletion mutants were particularly useful to address the regulatory roles of c-di-AMP in Gram-positive bacteria (29–31).

Here, we constructed and characterized a c-di-AMP PDE, *gdpP* deletion mutant in S. gallolyticus subsp. gallolyticus UCN34. We found that the S. gallolyticus subsp. gallolyticus UCN34 Δ*gdpP* mutant was morphologically smaller than the parental strain UCN34, more sensitive to osmotic stress, formed less biofilm on abiotic surfaces, attached less efficiently, and formed less cell aggregates on human intestinal cells. Furthermore, a genome-wide transcriptomic analysis indicated that c-di-AMP regulates many other important biological processes and modulates the expression of a few genes associated with pathogenicity. Overall, our results indicate that c-di-AMP could be an important signaling molecule controlling the ability of S. gallolyticus to colonize the host.

## RESULTS

### Deletion of *GALLO_2236* results in increased intracellular c-di-AMP levels in S. gallolyticus subsp. gallolyticus UCN34.

**FIG 1:**
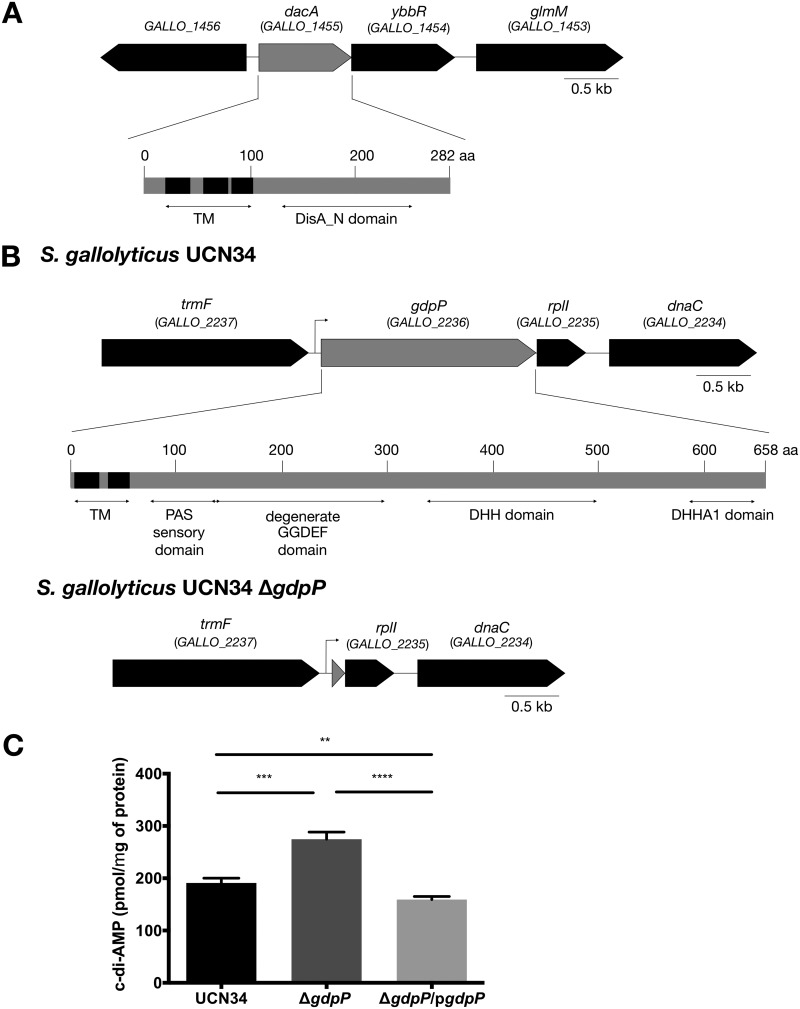
*GALLO_1455* and *GALLO_2236* encode c-di-AMP diadenylate cyclase and phosphodiesterase, respectively. (A and B) Gene locations and the domain architecture of GALLO_1455 (A) and GALLO_2236 (B) resemble the typical properties of DacA and GdpP, respectively. PAS, GGDEF, and DHH/DHHA1 domains were deleted to generate *S. gallolyticus* subsp. *gallolyticus* UCN34 Δ*gdpP*. Sixty-nine base pairs of the gene at the 3′ end of *gdpP* was left undeleted to preserve the ribosomal binding site of *rplI*. The arrow at the upstream of *GALLO_2236* marks the putative transcriptional start site of *GALLO_2236* based on *in silico* algorithm-based promoter prediction. (C) Liquid chromatograph-mass spectrometry (LC-MS) quantification of the intracellular concentration of c-di-AMP in *S. gallolyticus* subsp. *gallolyticus* UCN34, the Δ*gdpP* mutant, and the Δ*gdpP*/p*gdpP* complemented strain. Error bars represent the standard deviation of the measurements from three samples. Ordinary one-way analysis of variance (ANOVA test): **, *P* ≤ 0.01; ***, *P* ≤ 0.001; ****, *P* ≤ 0.0001.

In *Firmicutes*, c-di-AMP is commonly synthesized by diadenylate cyclase DacA and hydrolyzed by a specific phosphodiesterase, GdpP (21, 29). In many bacterial genomes, *dacA* often colocalizes with *ybbR* and *glmM*, whereas *gdpP* often colocalizes and is coexpressed with *rplI* and *dnaC* (18, 32). By protein homology search and gene location identification in the genome of S. gallolyticus subsp. gallolyticus UCN34, we identified *GALLO_1455* (GenBank accession number CBI13946) and *GALLO_2236* (GenBank accession number CBI14727) as the best candidates for *dacA* and *gdpP*, respectively (33). Further analysis of *GALLO_1455* and *GALLO_2236* in the SMART database (http://smart.embl-heidelberg.de) confirmed that GALLO_1455 and GALLO_2236 contain the typical domain architecture of DacA and GdpP, respectively (18, 34). In particular, GALLO_1455 contains three transmembrane regions and a DisA_N domain, whereas GALLO_2236 contains two transmembrane regions, a PAS sensory domain, a GGDEF domain, and a DHH/DHHA1 catalytic domain (Fig. 1A and B). We, therefore, renamed *GALLO_1455* and *GALLO_2236* as *dacA* and *gdpP*, respectively.

Notably, the S. gallolyticus subsp. gallolyticus UCN34 genome neither encodes a PgpH-type (19) nor a CdnP-type (14) c-di-AMP phosphodiesterase, but it does encode a stand-alone DHH/DHHA1 domain-containing protein (GALLO_0742). The stand-alone DHH/DHHA1 domain-containing protein, usually designated as Pde2 (or DhhP), was previously reported to hydrolyze c-di-AMP (35, 36) but has been shown recently to preferentially act on linear nucleotides, such as pApA and pGpG (36–38). In addition, Pde2 also exhibits additional enzymatic properties, such as being a nanoRNA RNase and a 3'-phosphoadenosine 5'-phosphate (pAp) phosphatase (36, 39, 40). Therefore, to investigate the roles of c-di-AMP in S. gallolyticus subsp. gallolyticus, the function of GALLO_0742 was not further explored in this study.

Our multiple attempts to create a *dacA* deletion mutant in S. gallolyticus subsp. gallolyticus UCN34 under standard laboratory culturing conditions by employing conventional knockout protocols were unsuccessful, suggesting that c-di-AMP may be essential for the survival of S. gallolyticus subsp. gallolyticus under standard culturing conditions, as has been shown for other Gram-positive bacteria (13, 24). Therefore, we focused on the construction of an in-frame *gdpP* deletion mutant to modulate the c-di-AMP levels in S. gallolyticus subsp. gallolyticus UCN34. We deleted the gene sequence encoding the PAS, the GGDEF, and the DHH/DHHA1 domains of GdpP and denoted this mutant strain S. gallolyticus subsp. gallolyticus UCN34 Δ*gdpP*. Of note, 69 bp at the 3′ end of *gdpP* was left undeleted to preserve the ribosomal binding site of the downstream gene *rplI* encoding ribosomal subunit L9 (Fig. 1B).

To verify that *gdpP* truly encodes a c-di-AMP phosphodiesterase in S. gallolyticus subsp. gallolyticus UCN34, we quantified the intracellular c-di-AMP levels of S. gallolyticus subsp. gallolyticus UCN34 wild type, the Δ*gdpP* mutant, and the *gdpP* complemented strain (Δ*gdpP*/p*gdpP*). As expected, the intracellular c-di-AMP levels of the Δ*gdpP* mutant were approximately 1.5-fold higher than in the wild-type strain UCN34. Importantly, the intracellular c-di-AMP levels in the Δ*gdpP*/p*gdpP* complemented strain were about 80% of the wild-type UCN34 (Fig. 1D), probably due to the overexpression of *gdpP* in the complemented strain.

### S. gallolyticus subsp. gallolyticus UCN34 Δ*gdpP* is morphologically smaller and is more sensitive to high osmotic stress than the parental strain.

**FIG 2:**
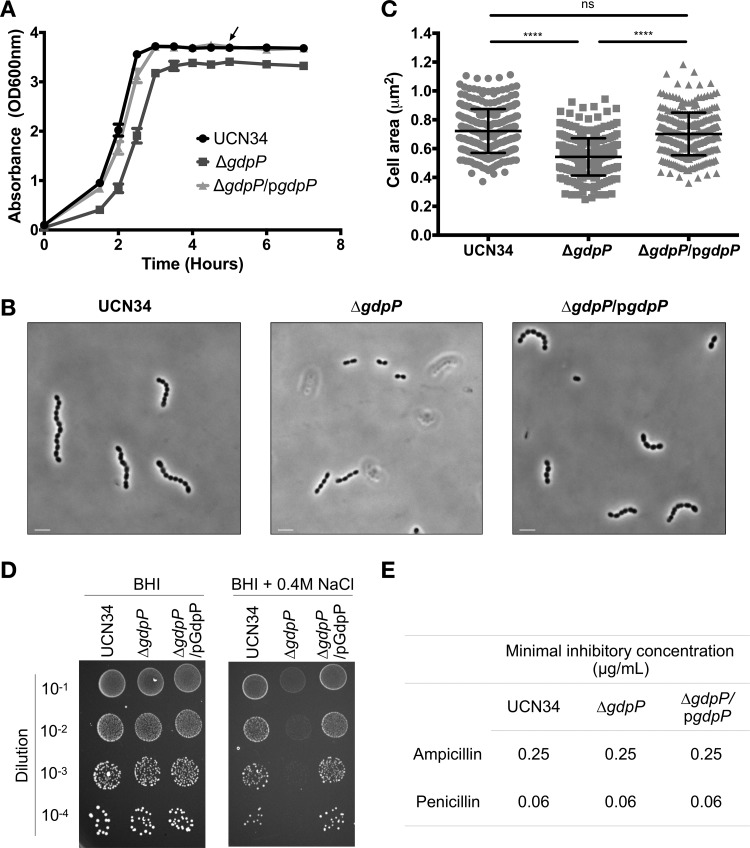
Phenotypic changes associated with an increased intracellular c-di-AMP levels resulted from the deletion of *gdpP*. (A) Representative anaerobic growth kinetics of *S. gallolyticus* subsp. *gallolyticus* UCN34, the Δ*gdpP* mutant, and the Δ*gdpP*/p*gdpP* complemented strain. Initial inoculum was prepared from log-phase culture adjusted to approximately 3 × 10^7^ CFU/ml. Growth, reflected in optical density, was measured at 600 nm (OD_600_) at the indicated time point. The arrow indicates the sample collection time point for biofilm assay and the RNA-seq experiment. Error bars represent the standard deviation of the measurements from three samples. (B) Representative phase-contrast microscopy images on the stationary-phase culture of the *S. gallolyticus* subsp. *gallolyticus* UCN34, the Δ*gdpP* mutant, and the Δ*gdpP*/p*gdpP* complemented strain. Images were acquired with Carl Zeiss Axio Observer.Z1 inverted wide-field microscope fitted with 100×/1.3-numerical-aperture (NA) objective oil lens. Images were processed using Imaris version 8.2. Scale bars = 3 μm. (C) Cell area measurement of 300 imaged cells from three independent experiments using ImageJ software. Error bars represent the standard deviation of the 300 measurements. Kruskal-Wallis test: ****, *P* ≤ 0.0001; ns, *P* > 0.05. (D) Representative images of 5 μl of *S. gallolyticus* subsp. *gallolyticus* log-phase culture adjusted to approximately 3 × 10^7^ CFU/ml spotted onto BHI agar and BHI agar supplemented with 0.4 M NaCl. (E) MICs of ampicillin and penicillin G against *S. gallolyticus* subsp. *gallolyticus* UCN34, the Δ*gdpP* mutant, and the Δ*gdpP*/p*gdpP* complemented strain. MIC was determined based on the optical density reading at 600 nm on a Tecan microplate reader, Infinite M200Pro.

We next investigated the physiological changes in the S. gallolyticus subsp. gallolyticus UCN34 Δ*gdpP* mutant in comparison to the wild type and the complemented strain. We first monitored the growth kinetics of S. gallolyticus subsp. gallolyticus UCN34, the Δ*gdpP* mutant, and the Δ*gdpP*/p*gdpP* complemented strain by measuring the optical density of the bacterial culture inoculated with the same number of cells (approximately 3 × 10^7^ CFU/ml). Throughout the experimental period, the optical density readings of the Δ*gdpP* mutant remained lower than that observed for the wild type and the Δ*gdpP*/p*gdpP* complemented strain (Fig. 2A). However, the doubling times of the three isogenic strains were similar (28.44 ± 2.16 min, 26.74 ± 2.23 min, and 30.86 ± 1.22 min for the wild type, the Δ*gdpP* mutant, and the Δ*gdpP*/p*gdpP* complemented strain, respectively; ±values are standard deviations [SD]) (see Data Set S1A in the supplemental material). A replotted growth curve based on CFU enumeration further showed that the increased intracellular c-di-AMP levels do not affect the growth of S. gallolyticus subsp. gallolyticus UCN34 (Data Set S1B).

When the bacterial cells were observed under phase-contrast microscopy, the UCN34 Δ*gdpP* mutant cells appeared clearly smaller than the wild type and the Δ*gdpP*/p*gdpP* complemented strain (Fig. 2B). A cell area measurement of 300 cells for each bacterial strain, using ImageJ software, confirmed that the UCN34 Δ*gdpP* mutant cells were about 25% smaller than the wild type and the Δ*gdpP*/p*gdpP* complemented strain (Fig. 2C). These data suggest that the lower optical density reading observed for the Δ*gdpP* mutant may primarily be due to the reduced cell size.

Increased intracellular c-di-AMP levels were previously reported to affect the bacterial tolerance to high osmotic stress and the sensitivity to β-lactam antibiotics (29, 41). Therefore, we tested the tolerance of S. gallolyticus subsp. gallolyticus UCN34, the Δ*gdpP* mutant, and the Δ*gdpP*/p*gdpP* complemented strain to high osmotic stress by spotting serially diluted log-phase culture on brain heart infusion (BHI) agar and BHI agar supplemented with 0.4 M NaCl. As shown in Fig. 2D, the S. gallolyticus subsp. gallolyticus UCN34 Δ*gdpP* mutant was more sensitive than the wild type to high osmotic stress. The tolerance to the osmotic stress was restored when *gdpP* was expressed in *trans*. We next tested the sensitivity of these 3 strains to β-lactam antibiotics. Our data did not show any significant differences in the susceptibility of the Δ*gdpP* mutant to ampicillin or penicillin G compared with that of the wild type and the complemented strain (Fig. 2E). Taken together, our data showed that high intracellular c-di-AMP affects bacterial cell size and tolerance to osmotic stress but not bacterial growth or sensitivity to β-lactam antibiotics in S. gallolyticus subsp. gallolyticus.

### Increased intracellular c-di-AMP levels reduce S. gallolyticus subsp. gallolyticus biofilm formation.

**FIG 3:**
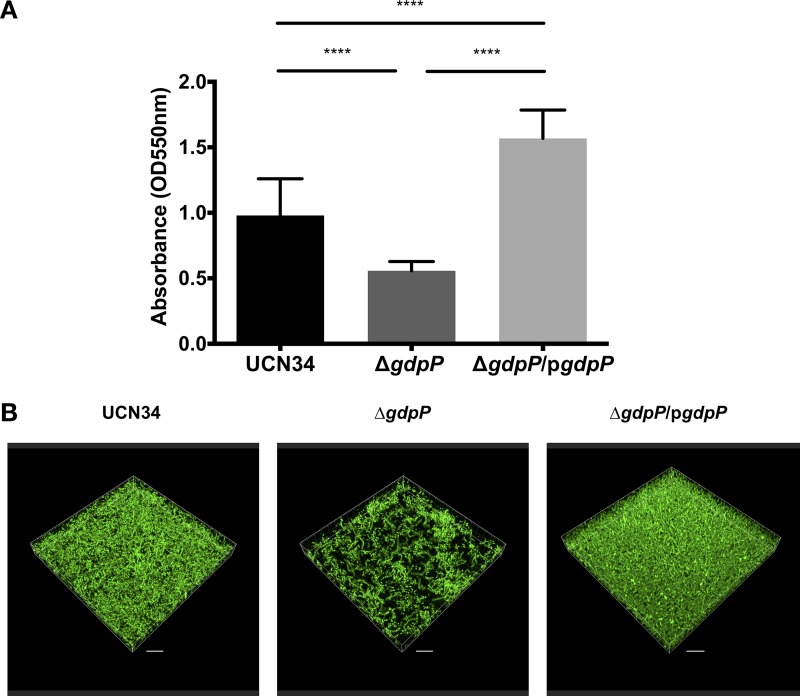
Accumulation of intracellular c-di-AMP levels inhibits *S. gallolyticus* subsp. *gallolyticus* biofilm formation. (A) Biofilm quantification using conventional microtiter plate biofilm assay. Error bars represent the standard deviation from 12 samples from 3 independent experiments. Ordinary one-way ANOVA test: ****, *P* ≤ 0.0001. (B) Representative biofilm images of *S. gallolyticus* subsp. *gallolyticus* UCN34, the Δ*gdpP* mutant, and the Δ*gdpP*/p*gdpP* complemented strain acquired using Carl Zeiss confocal laser scanning microscope LSM780 fitted with Plan Apochromat 100×/1.4-NA oil objective lens, with excitation at 488 nm. Scale bars = 10 μm.

c-di-AMP was previously shown to regulate biofilm formation in a number of Gram-positive bacteria (29–31, 42). To test the impact of increased intracellular c-di-AMP levels on S. gallolyticus subsp. gallolyticus biofilm formation, conventional biofilm assays on microtiter plates were carried out with S. gallolyticus subsp. gallolyticus UCN34, the Δ*gdpP* mutant, and the Δ*gdpP*/p*gdpP* complemented strain. Unexpectedly, we found that the biofilm formed by the Δ*gdpP* mutant was only 57% of the wild-type level, whereas the biofilm formed by the Δ*gdpP*/p*gdpP* complemented strain was 160% of the wild-type level (Fig. 3A). Confocal laser scanning microscopy images of the biofilms suggested that the reduced biofilm formation of the Δ*gdpP* mutant was primarily due to the reduced attachment of the Δ*gdpP* mutant cells to the surfaces (Fig. 3B). Taken together, our data indicate that biofilm formation of S. gallolyticus subsp. gallolyticus is negatively regulated by c-di-AMP, i.e., an increased intracellular c-di-AMP level reduces biofilm formation, whereas a decreased intracellular c-di-AMP level enhances biofilm formation.

### Increased intracellular c-di-AMP levels attenuate the ability of S. gallolyticus subsp. gallolyticus to adhere and to form cell aggregates on human colonic epithelial cells.

**FIG 4:**
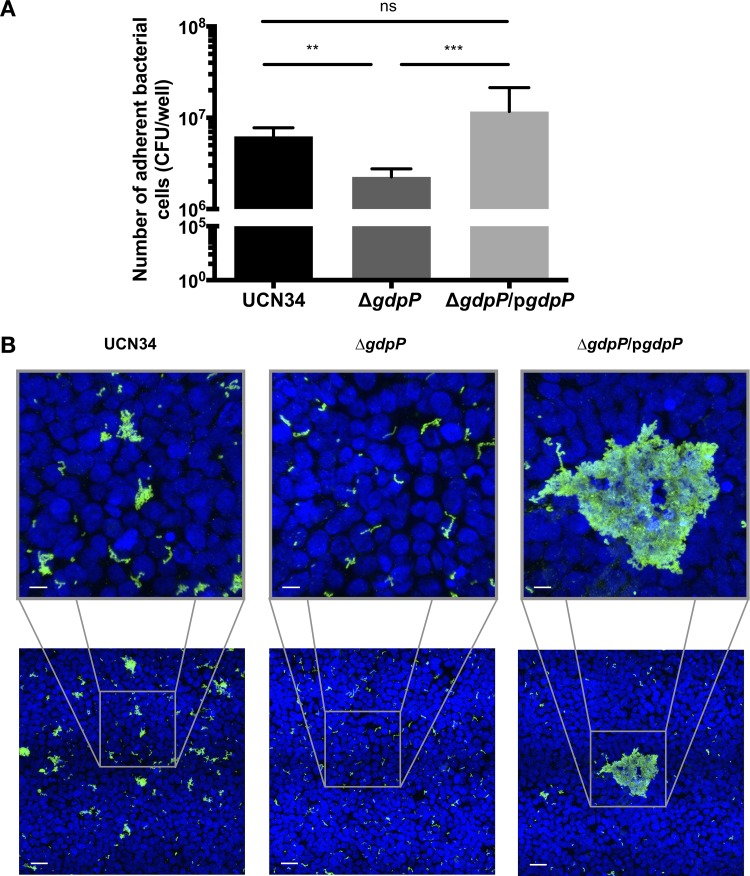
Increased intracellular c-di-AMP levels attenuate the ability of *S. gallolyticus* subsp. *gallolyticus* to adhere and to form cell aggregates on human colonic epithelial cells. (A) Quantification of the cell number of *S. gallolyticus* subsp. *gallolyticus* attached on a monolayer HT-29 human colorectal adenocarcinoma cells. Error bars represent the standard deviation of 9 samples from 3 independent experiments. Kruskal-Wallis test: **, *P* ≤ 0.01; ***, *P* ≤ 0.001; ns, *P* > 0.05. (B) Representative immunofluorescence images of the *S. gallolyticus* subsp. *gallolyticus* adhered on a monolayer of HT-29 human colorectal adenocarcinoma cells. Green, *S. gallolyticus* subsp. *gallolyticus* labeled with primary antibody rabbit UCN34 and secondary goat anti-rabbit antibody conjugated with Alexa Fluor 488. Blue, Hoechst 33342-stained DNA of the HT-29 cells. Scale bars = 10 μm (top) and 70 μm (bottom).

We next tested whether c-di-AMP can alter the ability of S. gallolyticus subsp. gallolyticus to adhere to biotic surfaces, such as human colonic cells. We introduced S. gallolyticus subsp. gallolyticus UCN34, the Δ*gdpP* mutant, and the Δ*gdpP*/p*gdpP* complemented strain onto a monolayer of human colorectal adenocarcinoma HT-29 cells and quantified the number of adherent bacterial cells after 1 hour of incubation at 37°C. As for biofilm formation, the Δ*gdpP* mutant adhered less efficiently on the monolayer of HT-29 cells than the wild type, whereas the complemented Δ*gdpP*/p*gdpP* strain adhered more efficiently than the wild type (Fig. 4A). Immunofluorescence microscopy showed that S. gallolyticus subsp. gallolyticus UCN34 formed cell aggregates on HT-29 cells, which was rarely observed for the Δ*gdpP* mutant. Interestingly, the complemented Δ*gdpP*/p*gdpP* strain formed larger cell aggregates than the wild type on HT-29 cells (Fig. 4B). These data demonstrated that c-di-AMP modulates the adherence of S. gallolyticus subsp. gallolyticus on both abiotic and biotic surfaces.

### c-di-AMP regulates various biological functions in S. gallolyticus subsp. gallolyticus UCN34, including gallocin production and Pil3 pilus biosynthesis.

To uncover other regulatory roles of c-di-AMP in S. gallolyticus subsp. gallolyticus, genome-wide transcriptomic sequencing (RNA-seq) was performed on the stationary phase culture of S. gallolyticus subsp. gallolyticus UCN34, the Δ*gdpP* mutant, and the Δ*gdpP*/p*gdpP* complemented strain. Comparative transcriptomic analysis revealed 109 genes whose expression was either upregulated or downregulated by ≥2-fold in the Δ*gdpP* mutant compared with the wild type and the complemented strain. Functional classification of these differentially regulated genes suggests that c-di-AMP regulates various biological functions, such as carbohydrate, amino acid, nucleotide, and coenzyme transport and metabolism; development of competence for genetic transformation; DNA replication, recombination, and repair; and translation, ribosomal structure, and biogenesis, in S. gallolyticus subsp. gallolyticus. In addition, several families of transcriptional regulators were found to be differentially expressed, which may serve as the mediators for c-di-AMP to regulate the cognate cellular functions (see Data Set S2 in the supplemental material).

Interestingly, the RNA-seq data also reflected that an increased intracellular c-di-AMP level was associated with the reduced expression of multiple ABC transporters, including spermidine/putrescine and proline/glycine betaine ABC transporters, which can be linked to osmotic regulation (Data Set S2). The S. gallolyticus subsp. gallolyticus UCN34 proline/glycine betaine ABC transporters are the homologs of the B. subtilis OpuCABCD osmoprotectant ABC transporters. In Staphylococcus aureus and L. monocytogenes, OpuCA containing a cystathionine-synthase (CBS) domain is a known c-di-AMP-binding protein (27, 43, 44). Whereas a putative homolog of OpuCA can be identified in S. gallolyticus subsp. gallolyticus UCN34 (GALLO_1283), GALLO_1283 appears to be a truncated OpuCA containing no CBS domain, which is similar to the OpuCA of Streptococcus pneumoniae (45). The CBS domain is required for c-di-AMP binding (43). Therefore, it is expected that c-di-AMP will not bind to the S. gallolyticus subsp. gallolyticus OpuCA to regulate the osmoprotectant transporter activity. Instead, as suggested by the RNA-seq data, c-di-AMP may control the uptake of osmoprotectant by regulating the transcription of the OpuCA transporter.

Intrigued by this observation, we further explored whether the putative homologs of other c-di-AMP-binding proteins were also differentially expressed in S. gallolyticus subsp. gallolyticus UCN34 Δ*gdpP*. These homologs include GALLO_2236 (homolog of PdeA) (46), GALLO_1832 (homolog of K^+^ transporter KtrA/KtrC/CabP/CabPA) (27, 31, 47–49), GALLO_1797 (homolog of K^+^ transporter CabPB) (27, 31), GALLO_1748 (homolog of P_II_-like signal transduction protein PstA) (46, 47, 50, 51), GALLO_1804 (homolog of hypothetical protein CbpB) (46), GALLO_1824 (homolog of transcriptional repressor NrdR) (46), and GALLO_2191 (homolog of recombination protein A RecA) (52). Notably, homologs of many other known c-di-AMP-binding proteins, such as cation/proton antiporter CpaA (46, 47, 53), sensor kinase KpdD (47, 54), transcriptional regulator BusR (27, 55) and DarR (56), pyruvate carboxylase PycA (46), hypothetical protein CbpA (46), and Lmo1466 (46), were not found in S. gallolyticus subsp. gallolyticus UCN34 (Table 1). None of these homologs, except the knocked out protein PdeA/GdpP, were differentially expressed in the S. gallolyticus subsp. gallolyticus Δ*gdpP* mutant compared with the wild type UCN34 and the complemented strain (Data Set S2). This result was not unexpected given that c-di-AMP interacts with the binding proteins to directly modulate the protein activity.

**TABLE 1:** Protein homologs of the known c-di-AMP-binding proteins in S. gallolyticus subsp. gallolyticus UCN34

Known c-di-AMP-binding protein	Homolog in UCN34	Differentially regulated by ≥2-fold in S. gallolyticus subsp. gallolyticus Δ*gdpP*?
Osmoprotectant transport ATP-binding protein OpuCA	GALLO_1283^*a*^	Yes
DHH subfamily 1 protein PdeA	GALLO_2236	Yes
K^+^ transporter KtrA/KtrC/CabP/CabPA	GALLO_1832	No
K^+^ transporter CabPB	GALLO_1797	No
P_II_-like signal transduction protein PstA	GALLO_1748	No
Hypothetical protein CbpB	GALLO_1804	No
Transcriptional repressor NrdR	GALLO_1824	No
Recombination protein A RecA	GALLO_2191^*b*^	No
Cation/proton antiporter CpaA	Not found	Not applicable
Sensor kinase KdpD	Not found	Not applicable
Transcriptional regulator BusR	Not found	Not applicable
Hypothetical protein Lmo1466	Not found	Not applicable
Pyruvate carboxylase PycA	Not found	Not applicable
Hypothetical protein CbpA	Not found	Not applicable
Transcriptional regulator DarR	Not found	Not applicable

a Homolog of OpuCA containing no cystathionine-synthase (CBS) domain.

b Does not contain the conserved motif of MsRecA for the binding of c-di-AMP.

**FIG 5:**
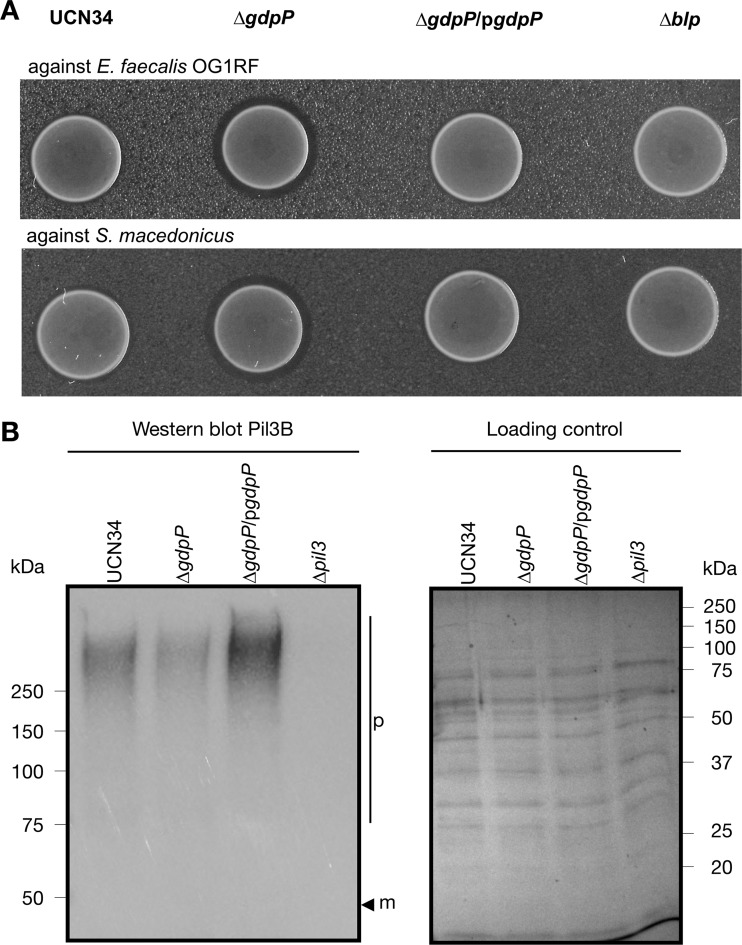
c-di-AMP regulates the gallocin production and Pil3 biosynthesis in *S. gallolyticus* subsp. *gallolyticus*. (A) Gallocin production by *S. gallolyticus* subsp. *gallolyticus* UCN34, the Δ*gdpP* mutant, and the Δ*gdpP*/p*gdpP* complemented strain under anaerobic conditions. Five microliters of *S. gallolyticus* subsp. *gallolyticus* UCN34, the Δ*gdpP* mutant, and the Δ*gdpP*/p*gdpP* complemented strain log-phase culture adjusted to approximately 3 × 10^7^ CFU/ml was spotted onto BHI agar flooded with Enterococcus faecalis OG1RF or *S. gallolyticus* subsp. *macedonicus*. Zone of clearance reflects the growth inhibition of E. faecalis or *S. gallolyticus* subsp. *macedonicus*. The strain deficient in producing the gallocin Δ*blp* mutant was used as the negative control. (B) Western blot analysis of the cell wall proteins from *S. gallolyticus* subsp. *gallolyticus* UCN34, the Δ*gdpP* mutant, and the Δ*gdpP*/p*gdpP* complemented strain. Equal amounts of the cell wall proteins were loaded (right) and probed with specific polyclonal antibodies against Pil3B (left). Cell wall proteins of the *S. gallolyticus* subsp. *gallolyticus* UCN34 Δ*pil3* mutant was used as a negative control. Theoretical positions of Pil3B monomers, based on the molecular weights, are indicated (m), and high-molecular-weight species corresponding to pilus polymers are labeled (p).

Importantly, the transcription of the *blpB* encoding gallocin and the *blpC* encoding gallocin immunity protein (10) was upregulated (∼2 fold), whereas the expression of *pil3A*, *pil3B*, and *srtC* of the Pil3 operon (12) was downregulated (∼4 fold) in the Δ*gdpP* mutant compared with the wild type and the complemented strain (Data Set S2). Gallocin and Pil3 pilus were previously shown as two important colonization factors enabling S. gallolyticus subsp. gallolyticus to persist in the murine colon (10, 12). The RNA-seq data indicating that these colonization factors are differentially expressed in the Δ*gdpP* mutant prompted us to assess the gallocin production and Pil3 pilus biosynthesis in the three isogenic S. gallolyticus subsp. gallolyticus strains. To measure gallocin production, we spotted approximately 2 × 10^5^ log-phase cells of UCN34, the Δ*gdpP* mutant, and the Δ*gdpP*/p*gdpP* complemented strain onto BHI agar flooded with the gallocin-sensitive strain Enterococcus faecalis OG1RF and S. gallolyticus subsp. macedonicus (10). The S. gallolyticus subsp. gallolyticus UCN34 Δ*blp* mutant producing no gallocin was used as a negative control (10). After an overnight incubation under anaerobic conditions, the S. gallolyticus subsp. gallolyticus UCN34 Δ*gdpP* mutant created a larger zone of inhibition than the wild type and the Δ*gdpP*/p*gdpP* complemented strain, demonstrating an increased gallocin production in the Δ*gdpP* mutant (Fig. 5B). To quantify Pil3 biosynthesis, Western blot analysis was carried out using the cell wall proteins from UCN34, the Δ*gdpP* mutant, and the Δ*gdpP*/p*gdpP* complemented strain. We included the isogenic UCN34 Δ*pil3* to check for antibody specificity. Our data showed that Pil3 expression was noticeably decreased in the Δ*gdpP* mutant compared with the wild type and the Δ*gdpP*/p*gdpP* complemented strain (Fig. 5C). Taken together, these data indicate that c-di-AMP modulates gallocin and Pil3 levels in S. gallolyticus subsp. gallolyticus.

## DISCUSSION

Streptococcus gallolyticus subsp. gallolyticus is an emerging pathogen responsible for septicemia and endocarditis in the elderly, whose presence is strongly associated with the occurrence of colorectal cancer (7, 8, 57–60). Here, we investigated the roles of c-di-AMP in this emerging pathogen. We show that c-di-AMP plays a pleiotropic role in S. gallolyticus subsp. gallolyticus, controlling the tolerance to osmotic stress, cell size, biofilm formation, adherence to intestinal cells, cell aggregate formation, gallocin production, and Pil3 pilus expression.

c-di-AMP has been proposed to regulate several processes in Gram-positive bacteria. One of the conserved roles is to maintain osmotic homeostasis (41). We speculate that c-di-AMP maintains this conserved role in S. gallolyticus subsp. gallolyticus for two reasons. First, the observations that the S. gallolyticus subsp. gallolyticus Δ*gdpP* mutant is more sensitive to osmotic stress and exhibits altered bacterial morphology suggest an underlying perturbation in the cellular turgor to osmotic pressure (28, 41, 61). Second, our RNA-seq data suggest that at high intracellular c-di-AMP levels, the expression of spermidine/putrescine and proline/betaine glycine ABC transporters was downregulated in S. gallolyticus subsp. gallolyticus. These transporters are crucial for bacterial cells for rapid osmotic adjustment during osmotic shift (62, 63). Combined, these data point toward a role of c-di-AMP in maintaining osmotic homeostasis in S. gallolyticus subsp. gallolyticus. Another proposed role of c-di-AMP is to indirectly mediate sensitivity to β-lactam antibiotics (41). However, we did not observe a significant difference in the MIC of β-lactam antibiotics across our wild type, the Δ*gdpP* mutant, and the Δ*gdpP*/p*gdpP* complemented strain. Similar observations were also reported in Streptococcus suis (30), suggesting that c-di-AMP may not mediate the sensitivity to β-lactam antibiotics in *Streptococcus* spp.

Notably, an increased intracellular c-di-AMP level reduces biofilm formation in S. gallolyticus subsp. gallolyticus. A similar finding was reported in B. subtilis (42). This result is in contrast to several other publications showing the opposite effect of c-di-AMP on the biofilm formation of S. aureus, S. suis, and Streptococcus mutans (29–31). The detailed mechanisms explaining how c-di-AMP affects the biofilm formation in S. gallolyticus subsp. gallolyticus are currently being investigated. It is important to emphasize that both S. gallolyticus subsp. gallolyticus and bacterial biofilms can play a role in the development of colorectal cancer (9, 64–67). It would be interesting to explore whether the presence of S. gallolyticus subsp. gallolyticus biofilms plays a role as the potential promoting factor in the development of colorectal cancer, and this can be approached by manipulating the intracellular c-di-AMP levels to modulate the biofilm formation of S. gallolyticus subsp. gallolyticus.

It is also worth mentioning that although the Pil3 pilus is an important colonization factor for S. gallolyticus *in vivo* in the murine colon, it plays a minor role in mediating the adherence of S. gallolyticus on HT-29 cells *in vitro* (12). Therefore, the reduced expression of Pil3 pilus in the S. gallolyticus subsp. gallolyticus Δ*gdpP* mutant may not solely account for the reduced adherence of the S. gallolyticus subsp. gallolyticus Δ*gdpP* mutant on HT-29 cells. We speculate that in the Δ*gdpP* mutant, the combined effects of the reduced expression of Pil3 pilus and of other putative adhesins may instead account for the reduced adherence on HT-29 cells and the absence of cell aggregates. Given that cell aggregates may progressively develop into biofilms (68), it awaits further investigation on whether Pil3 pilus and the putative adhesins establish a link between biofilm formation, cell attachment, cell aggregation, and potentially host colonization in S. gallolyticus subsp. gallolyticus.

In this study, despite a relatively low difference in the intracellular c-di-AMP levels in the S. gallolyticus subsp. gallolyticus Δ*gdpP* mutant and the Δ*gdpP*/p*gdpP* complemented strain compared with the wild type, significant phenotypic differences were observed especially in terms of biofilm formation and adherence on intestinal cells. Small differences in the intracellular c-di-AMP were also observed for S. pneumoniae, S. suis, and B. subtilis and their isogenic *gdpP* mutants (16, 30). In sharp contrast, S. aureus and S. agalactiae *gdpP* mutants were shown to accumulate up to 38-fold more intracellular c-di-AMP than the parental strains (27, 29). The driving factors for the difference are currently unclear.

In conclusion, we report here that the second messenger signaling molecule c-di-AMP controls osmotic tolerance, biofilm formation on abiotic surfaces, adherence on human intestinal cells, formation of cell aggregates, expression of pilus proteins, and production of bacteriocin in S. gallolyticus subsp. gallolyticus. This study, thus, indicates that c-di-AMP could be an important signaling molecule governing the pathogenicity of S. gallolyticus subsp. gallolyticus.

## MATERIALS AND METHODS

### Bacterial strains, culturing conditions, plasmids, and primers.

All bacterial strains, plasmids, and primers used in this study are listed in Table 2. Unless stated otherwise, an overnight culture of S. gallolyticus subsp. gallolyticus UCN34 was typically prepared from a single colony in 5 ml of M9YEG broth (1× M9 minimal salts [MP Biomedicals] supplemented with 0.5% of yeast extract [Becton, Dickinson, and Company] and 1.0% glucose [VWR]) and incubated at 37°C under static conditions for 12 to 14 hours. The overnight culture was diluted (1:5) in M9YEG broth the next day and incubated further at 37°C for the preparation of log-phase culture. When necessary, erythromycin was supplemented to a final concentration of 2 μg/ml (Sigma-Aldrich).

**TABLE 2:** Bacterial strains, plasmids, and primers used in this study

Strain, plasmid, or primer	Description	Reference or source
Strains		
E. coli DH5α	*deoR endA1 gyrA96 hsdR17* (Δ*lac*)*U169 recA1 relA1 supE44 thi-1* (ϕ80 *lacZ*ΔM15)	Lab collection
S. gallolyticus subsp. gallolyticus UCN34	A clinical strain isolated from an infective endocarditis patient who later diagnosed with CRC^*a*^	33
UCN34 Δ*gdpP*	In-frame *gdpP* (*GALLO_2236*) knockout mutant	This study
UCN34 Δ*gdpP*/p*gdpP*	*gdpP* complemented strain; UCN34 Δ*gdpP* containing pTCV*erm::gdpP*	This study
UCN34 Δ*pil3*	In-frame *pil3* (*GALLO_2038* to *GALLO_2042*) knockout mutant	12
UCN34 Δ*blp*	Gallocin-deficient knockout mutant (*GALLO_2021* to *GALLO_2020*)	10
S. agalactiae NEM316	MLST-23, serotype III isolated from neonate blood culture	73
E. faecalis OG1RF	Derived from a clinical isolate OG1; rifampicin and fusidic acid resistant	From Kimberly Kline
Plasmids		
pG1	Em; *oriR* pUC; *oriR*(Ts) pWV01; MCS pUC18	72
pG1::*gdpP*KO	pG1 containing 2-kb fragment corresponding to the 5′ and 3′ end of *GALLO_2236*	This study
pTCV*erm-oriT_TnGBS1_*	Em Km Mob^+^ (IncP); *oriR* pACYC184; *oriR* pAMβ1; MCS *lacZ*α^+^	72
pTCV*erm::gdpP*	pTCV*erm-oriT_TnGBS1_* containing promoter and ORF of *gdpP* (*GALLO_2236*)	This study
Primers		
gdpP_up_F	GTCAAACCAATGGTACG	This study
gdpP_up_R	TAAGTGTTCGGCTTGACTCAAGCCTATCATGACTAA	This study
gdpP_dn_F	TTAGTCATGATAGGCTTGAGTCAAGCCGAACACTTA	This study
gdpP_dn_R	CTGCGATTGCTATGTTC	This study
gdpP_F	CACAGGATCCGTTTACCTAGAAGGCAAG^*b*^	This study
gdpP_R	CACAGCATGCCATCACTCTACCTCCAT^*b*^	This study

a CRC, colorectal cancer.

b Restriction sites are underlined.

### Construction of *gdpP* deletion mutant.

The construction of the *gdpP* in-frame deletion mutant was performed as previously described (69, 70). In brief, two approximately 1-kb DNA fragments corresponding to the 5′ and 3′ end of *gdpP* were PCR-amplified using primer pairs gdpP_up_F/gdpP_up_R and gdpP_dn_F/gdpP_dn_R. The resulting PCR products were purified and further amplified using primer pair gdpP_up_F and gdpP_dn_R and subsequently cloned into pG1 plasmid, generating pG1::*gdpP*KO. pG1::*gdpP*KO was introduced into S. agalactiae NEM316 (71) by electroporation and later into S. gallolyticus subsp. gallolyticus UCN34 by conjugal transfer. S. gallolyticus subsp. gallolyticus UCN34 with pG1::*gdpP*KO integrated into the genome was selected by growing the bacteria at 37°C in the presence of erythromycin. Next, integrants were serially passaged at 30°C in BHI broth without antibiotic to facilitate the excision of the plasmid by homologous recombination. An in-frame deletion of *gdpP* gene was verified by PCR and Sanger sequencing of the *gdpP*-chromosomal flanking regions.

### Construction of *gdpP* complemented strain.

A DNA fragment containing the putative promoter and the full-length open reading frame of *GALLO_2236* was amplified by PCR using the primer pair gdpP_F and gdpP_R. The PCR product was restricted with BamHI and SphI and ligated to BamHI*/*SphI-restricted pTCV*erm-oriT_TnGBS1_*, generating a *GALLO_2236* complementation plasmid, pTCV*erm*::*gdpP*. The complementation plasmid was introduced into Escherichia coli DH5α and then extracted and sequenced. Next, it was introduced into S. agalactiae NEM316 and later into S. gallolyticus subsp. gallolyticus UCN34 Δ*gdpP*, as described earlier (70).

### Preparation of c-di-AMP extract.

Intracellular c-di-AMP was extracted following the published protocol, with minor modifications (29). A total of 6 ml of log-phase culture adjusted to approximately 3 × 10^7^ CFU/ml was added into each well of a 6-well plate. After 5 hours of incubation at 37°C under anaerobic conditions (0% O_2_, 10% CO_2_; AnaeroGen Compact, prepared according to the manufacturer’s instruction; Thermo Fisher Scientific), the bacterial culture was well mixed by using cell scrapers (TPP) and repeated pipetting. A total of 300 μl of the well-mixed bacterial culture was collected, pelleted, lysed in 300 μl of 0.1 M NaOH for 10 minutes at 80°C, and was subjected to protein quantification with a Qubit 2.0 fluorometer (Thermo Fisher Scientific) for normalization purposes. Five milliliters of the well-mixed bacterial culture was transferred to a 15-ml Falcon tube and centrifuged at 8,000 × *g* for 2 minutes at 4°C. The bacterial pellet was washed once with 0.9% NaCl. One milliliter of ice-cold extraction buffer (40% [vol/vol] acetonitrile, 40% [vol/vol] methanol, and 20% ultrapure water) was added to the pellet, and the suspension was mixed well. The samples were snap-frozen in liquid nitrogen for 30 seconds before being boiled for 10 minutes. The samples were subsequently transferred to Lysing Matrix B tubes (MP Biomedicals) and were homogenized in a FastPrep-24 instrument at a setting of 6.0 m/s for 45 seconds (MP Biomedicals) before being separated from the silica beads by centrifugation at 17,000 × *g* for 5 minutes at 4°C. A total of 600 μl of the top layer was transferred to a new tube. The silica beads/cell debris mixture was added with 1 ml of ice-cold extraction buffer, briefly vortexed, and incubated on ice for 5 minutes, before a second centrifugation at the same setting. The top layer was again collected and combined with the first extract. The samples were dried at 4°C in a CentriVap centrifugal vacuum concentrators (Labconco). Dried samples were stored at −80°C until analysis.

### Quantification of c-di-AMP extract by liquid chromatography-mass spectrometry analysis.

Detection and quantification of c-di-AMP were performed as described previously with modifications (72) at the Singapore Phenome Centre. Briefly, it was performed with a Xevo TQ-S instrument (Waters) with a binary pump, a temperature-controlled autosampler maintained at 4°C, and a column oven compartment maintained at 40°C, interfaced to the electrospray ionization (ESI) positive ion source. A total of 5 μl of the c-di-AMP extract dissolved in 100 μl of water was injected into a BEH C_18_ column (1.7 μm; 2.1 by 50 mm; Waters). Mobile phase A was 10 mM ammonium formate in water containing 0.1% formic acid, whereas mobile phase B was methanol containing 0.1% formic acid. Samples were run in gradient condition, with 100% mobile phase A from initial to 3 minutes, 80% mobile phase A from 3 to 3.5 minutes, 10% mobile phase A from 3.5 to 6.5 minutes, and 100% mobile phase A from 6.6 to 8 minutes. The total run was 8 minutes, with a flow rate of 0.30 ml per minute. Software MassLynx and TargetLynx were used for chromatography and quantification of c-di-AMP, respectively.

### Growth curve of S. gallolyticus subsp. gallolyticus UCN34 and derivatives.

A log-phase S. gallolyticus subsp. gallolyticus culture was diluted to approximately 3 × 10^7^ CFU/ml in M9YEG broth. One milliliter of the culture was seeded into each well of a 24-well plate and incubated at 37°C under anaerobic conditions. The bacterial growth was monitored at the desired time point by optical density (OD) measurement at 600 nm using a UV spectrophotometer.

### Antibiotic susceptibility test.

Ampicillin and penicillin G (Sigma-Aldrich) dissolved in water were 2× serially diluted in M9YEG broth in a 96-well microtiter plate. One hundred microliters of log-phase S. gallolyticus subsp. gallolyticus culture diluted to approximately 1 × 10^6^ CFU/ml was added into the wells containing antibiotics. After a 20-hour incubation, the plate was measured at a wavelength of 600 nm on a microplate reader, Infinite M200Pro (Tecan). The lowest concentration of antibiotics that inhibited the bacterial growth was determined as the MIC.

### Microtiter plate biofilm assay.

The assay was performed as described with modifications (73). One milliliter of log-phase S. gallolyticus subsp. gallolyticus culture diluted to approximately 3 × 10^7^ CFU/ml was added into each well of a 24-well plate and incubated at 37°C under anaerobic conditions (0% O_2_, 10% CO_2_; AnaeroGen Compact, prepared according to the manufacturer’s instruction; Thermo Fisher Scientific). After 5 hours of incubation, the bacterial culture was removed. The wells were washed twice with 0.9% NaCl, before being stained with 1 ml of 0.1% of crystal violet solution for 15 minutes. Following this step, the crystal violet solution was removed, the wells were washed twice with 0.9% NaCl, and the biofilm was solubilized by 30% acetic acid for 15 minutes. Solubilized biofilm was quantified and measured on a microplate reader, Infinite M200Pro (Tecan), at a wavelength of 550 nm.

### Confocal laser scanning microscopy imaging.

The 5-hour biofilms formed by S. gallolyticus subsp. gallolyticus UCN34 and the derivatives under anaerobic conditions were washed with 0.9% NaCl twice, fixed with 4% paraformaldehyde for 10 minutes, and stained with SYTO9 (1:500 diluted from stock; Thermo Fisher Scientific) for 10 minutes. Biofilm images were acquired using LSM780 inverted confocal laser scanning microscope (Carl Zeiss) fitted with Plan Apochromat 100×/1.4-numerical-aperture (NA) oil objective lens, with excitation at 488 nm. The images were processed using Imaris version 8.2.0 (Bitplane).

### Cell adherence assay.

The human colorectal adenocarcinoma cell line ATCC HTB-38 (HT-29) was routinely maintained in Dulbecco modified Eagle medium (DMEM) high glucose (with l-glutamine, without sodium pyruvate; Gibco) supplemented with 10% fetal bovine serum. One milliliter of the log-phase S. gallolyticus subsp. gallolyticus culture diluted to approximately 5 × 10^7^ CFU/ml was seeded onto a monolayer of HT-29 cells cultured in a 24-well plate, with a multiplicity of infection of 20. After 1 hour of incubation at 37°C and 5% CO_2_, the monolayer was washed two times with phosphate-buffered saline (PBS) to remove the nonadherent bacteria. The monolayer was resuspended in 0.05% Triton X-100. The amount of adherent bacteria was determined by CFU count.

### Immunofluorescence imaging.

A monolayer of HT-29 cells was infected as described above in “Cell adherence assay.” Following incubation, the monolayer was washed once with PBS and fixed in 4% paraformaldehyde for 15 minutes. The samples were subsequently incubated for 1 hour in PBS containing rabbit anti-UC34 (1:200), followed by an additional 1-hour incubation in PBS containing Alexa Fluor 488-conjugated goat anti-rabbit antibody (1:200; Abcam) and Hoechst 33342 (1:500; Thermo Fisher Scientific) (10). The samples were imaged using an LSM780 inverted confocal laser scanning microscope (Carl Zeiss) fitted with Plan Apochromat 40×/1.3-NA and 63×/1.4-NA oil objective lenses, with excitation at 405 nm and 488 nm. Tile scan images were stitched using Image Stitching plug-ins on Fiji (74). All images were processed using Imaris version 8.2.0 (Bitplane).

### RNA extraction and sequencing.

One milliliter of the log-phase S. gallolyticus subsp. gallolyticus culture diluted to approximately 3 × 10^7^ CFU/ml was added into each well of a 24-well plate and incubated at 37°C under anaerobic conditions (0% O_2_, 10% CO_2_; AnaeroGen Compact, prepared according to the manufacturer’s instruction; Thermo Fisher Scientific). After 5 hours of incubation, the bacterial culture was removed and preserved in 2 volumes of RNAprotect bacterial reagent (Qiagen) and extracted using an RNeasy minikit (Qiagen) according to the manufacturer’s instruction. The extracted RNA was depleted by using a Ribo-Zero rRNA removal kit (Bacteria) (Illumina) and converted to cDNA by using the NEBNext RNA first strand synthesis module and NEBNext Ultra directional RNA second strand synthesis module (New England Biolabs) and subsequently sequenced on the Illumina HiSeq 2500 platform (100-bp paired-end reads) in our in-house sequencing facility.

### RNA-seq data analysis and functional annotation.

The sequencing raw reads from the RNA-seq experiment were trimmed and mapped to the S. gallolyticus subsp. gallolyticus UCN34 genome with CLC Genomics Workbench 8.0. A differential analysis of the S. gallolyticus subsp. gallolyticus UCN34, the Δ*gdpP* mutant, and the Δ*gdpP*/p*gdpP* complemented strain was performed using the R/Bioconductor DEseq2 package. Functional annotation on the differentially regulated genes was performed based on Clusters of Orthologous Groups (COG) classification and manually corrected based on published literatures.

### Cell wall extract preparation and immunoblotting.

Cell wall extracts were prepared as described earlier (75) and quantified using a Qubit 2.0 fluorometer (Thermo Fisher Scientific). Equal amounts of the cell wall extracts were boiled in NuPAGE lithium dodecyl sulfate (LDS) sample buffer (Thermo Fisher Scientific), separated by SDS-PAGE on a NuPAGE 4 to 12% bis-Tris protein gradient gel (Thermo Fisher Scientific), and transferred to a polyvinylidene difluoride (PVDF) membrane using an iBlot transfer pack (Thermo Fisher Scientific). The membrane was blocked in casein blocking buffer (Sigma-Aldrich) and incubated for 1 hour with rabbit primary Pil3B antibodies (1:1,000) and subsequently with horseradish peroxidase-conjugated goat anti-rabbit antibody (1:5,000). The membrane was washed 3 times with PBS and 0.1% Tween 20 between the incubation with antibodies. Chemiluminescence was detected on a ChemiDoc gel imaging system (Bio-Rad Laboratories).

### Accession number(s).

The raw RNA-seq reads were deposited at the NCBI Sequence Read Archive (SRA) database under BioProject number PRJNA484077.

## Supplementary Material

Supplemental file 1
