# Supplementary material for: Increased Intracellular Cyclic di-AMP Levels Sensitize Streptococcus gallolyticus subsp. gallolyticus to Osmotic Stress and Reduce Biofilm Formation and Adherence on Intestinal Cells
Source: J Bacteriol. 2019 Feb 25;201(6):e00597-18. doi: 10.1128/JB.00597-18 (PMC6398277; doi:10.1128/JB.00597-18)
Supplement: Supplemental file 1 [file JB.00597-18-s0001.pdf]

A

	Doubling time (min)
UCN34	28.44 ± 2.16
Δ*gdpP*	26.74 ± 2.23
Δ*gdpP*/p*gdpP*	30.86 ± 1.22

B

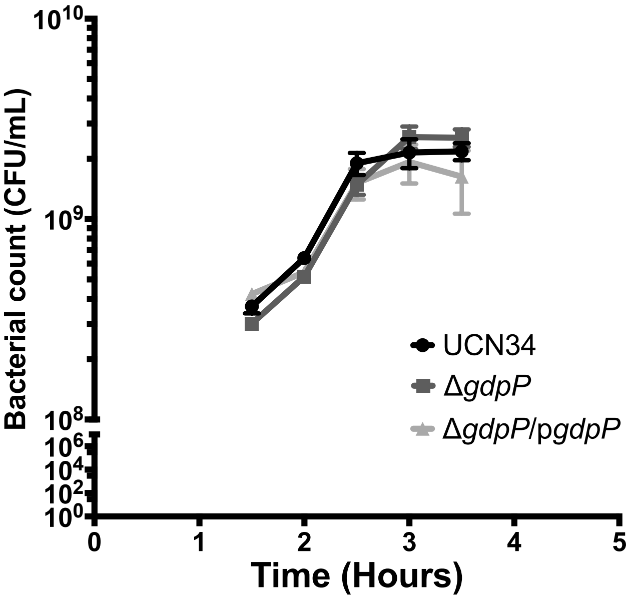

Supplemental Data S1. High intracellular c-di-AMP does not significantly affect the growth of *S. gallolyticus* UCN34. (A) Doubling time of *S. gallolyticus* UCN34, the Δ*gdpP* mutant and the Δ*gdpP*/p*gdpP* complemented strain was calculated based on the absorbance value of samples from 3 independent experiments. (B) Growth curve of *S. gallolyticus* UCN34, the Δ*gdpP* mutant and the Δ*gdpP*/p*gdpP* complemented strain plotted based on CFU enumeration at the indicated time point.

Functions	Locus tag	Gene annotation	Δ*gdpP* vs UCN34	Δ*gdpP* vs Δ*gdpP*/p*gdpP*
Up-regulation				
Transcription	Gallo_0126	Putative transcriptional regulator, MutR family	2.2203	2.0186
Transcription	Gallo_0876	Putative transcriptional regulator, GntR family	2.1961	2.1038
Transcription	Gallo_1206	Putative transcriptional regulator, AraC family	2.4988	2.7743
Transcription	Gallo_1524	Putative transcriptional regulator, TetR family	3.7866	4.8320
Transcription	Gallo_2146	Putative phage transcriptional regulator	2.2095	2.1041
Transcription	Gallo_2208	Putative transcriptional regulator	2.3114	2.0343
Gallocin production	Gallo_2019	BlpC, gallocin-immunity protein	2.4953	3.5185
Gallocin production	Gallo_2020	BlpB, gallocin	2.0721	3.4750
Competence for genetic transformation	Gallo_0087	Putative competence protein ComGA	4.6259	3.1731
Competence for genetic transformation	Gallo_0088	Putative competence protein ComGB	3.0641	3.0039
DNA replication, recombination and repair	Gallo_0833	Putative 7,8-dihydro-8-oxoguanine-triphosphatase (MutT)	2.1296	2.0645
DNA replication, recombination and repair	Gallo_0627	Putative exonuclease VII large subunit	2.6346	2.3805
DNA replication, recombination and repair	Gallo_0628	Putative exonuclease VII small subunit	2.4982	2.0377
Translation, ribosomal structure and biogenesis	Gallo_0128	Putative ribosomal large subunit pseudouridine synthase, RluD family	2.4465	2.3700
Translation, ribosomal structure and biogenesis	Gallo_1418	GuaD; Putative guanine deaminase	2.6843	2.4553
Translation, ribosomal structure and biogenesis	Gallo_2260	Conserved hypothetical protein	2.2551	2.8034
Carbohydrate transport and metabolism	Gallo_0153	Putative PTS system, lactose/cellobiose-specific, EIIB component	2.7401	2.1511
Carbohydrate transport and metabolism	Gallo_0961	Putative glycosyl hydrolase	2.3043	2.0720
Carbohydrate transport and metabolism	Gallo_1055	GtfA; Putative glucosyltransferase	2.5654	3.9848
Carbohydrate transport and metabolism	Gallo_0238	Phosphoglycerate mutase family protein	2.3446	2.2999
Carbohydrate transport and metabolism	Gallo_1460	GlxK; Putative glycerate kinase	2.0648	2.0631
Coenzyme transport and metabolism	Gallo_0218	Putative HAD-superfamily hydrolase/phosphatase	2.0432	2.3437
Coenzyme transport and metabolism	Gallo_0362	Conserved hypothetical protein	2.3278	2.0179
Nucleotide transport and metabolism	Gallo_0025	PurC; phosphoribosylaminoimidazole succinocarboxamide synthetase	2.3515	2.3036
Amino acid transport and metabolism	Gallo_1809	LysA; diaminopimelate decarboxylase	2.1265	2.5204
Unknown	Gallo_0577	Putative collagen-binding protein, peptidoglycan linked protein (LPXTG motif)	3.7235	4.4586
Unknown	Gallo_0696	Putative serine-rich lipoprotein	4.9980	3.8973
Unknown	Gallo_1689	Tn916 conserved hypothetical protein	2.0751	2.0279
Unknown	Gallo_1690	Tn916 conserved hypothetical protein	2.3340	2.1500
Unknown	Gallo_1691	Tn916 conserved hypothetical protein	2.0016	2.1037
Unknown	Gallo_1692	Tn916 conserved hypothetical protein	2.0663	2.3064
Unknown	Gallo_1693	Tn916 conserved hypothetical protein	2.0395	2.4673
Unknown	Gallo_1694	Tn916 conserved hypothetical protein	2.2405	2.2777
Unknown	Gallo_1695	Tn916 conserved hypothetical protein	2.5377	2.4170
Unknown	Gallo_1696	Putative transcriptional regulator, Cro/CI family (Tn916)	2.2828	2.6422
Unknown	Gallo_1697	Tn916 conserved hypothetical protein, putative translocase	2.1169	2.2720
Unknown	Gallo_1698	Tn916 conserved hypothetical protein	2.0943	2.4597
Unknown	Gallo_1701	Conserved hypothetical protein	2.4449	2.5155
Unknown	Gallo_1702	Conserved hypothetical protein	2.3774	2.5283
Unknown	Gallo_0129	Conserved hypothetical protein	3.0382	2.2538
Unknown	Gallo_0275	Conserved hypothetical protein, transposon related	2.5606	2.3988
Unknown	Gallo_0276	Hypothetical protein	2.0167	2.7539
Unknown	Gallo_0277	Hypothetical protein	2.0118	2.3777
Unknown	Gallo_0298	Hypothetical protein	2.5655	2.8552
Unknown	Gallo_0401	Conserved hypothetical protein	2.2383	2.1381
Unknown	Gallo_0553	Conserved hypothetical protein	2.2815	2.3283
Unknown	Gallo_0578	Conserved hypothetical protein	2.7291	2.3280
Unknown	Gallo_0579	Conserved hypothetical protein	2.2829	3.3717
Unknown	Gallo_0580	Conserved hypothetical protein	7.9744	6.4795
Unknown	Gallo_0610	Conserved hypothetical protein	2.6511	2.0823
Unknown	Gallo_0611	Conserved hypothetical protein, MutT/nudix family	2.4904	3.8769
Unknown	Gallo_0647	Conserved hypothetical protein	2.4801	2.5021
Unknown	Gallo_1066	Conserved hypothetical protein	2.3822	2.3172
Unknown	Gallo_1199	Hypothetical protein	2.1046	2.6831
Unknown	Gallo_1526	Conserved hypothetical protein	2.4440	3.4303
Unknown	Gallo_1704	Conserved hypothetical protein	2.1550	2.5287
Unknown	Gallo_1705	Conserved hypothetical protein	2.4121	2.0303
Unknown	Gallo_1943	Conserved hypothetical protein	2.2780	2.0434
Unknown	Gallo_1951	Conserved hypothetical protein	2.0056	2.1246
Unknown	Gallo_1955	Conserved hypothetical protein	2.4103	2.0387
Unknown	Gallo_2215	Conserved hypothetical protein	2.3301	2.6427
Down-regulation				
Signal transduction mechanisms	Gallo_2236	GdpP; c-di-AMP phosphodiesterase	-63.2552	-982.8238
Ligands transportation across membrane	Gallo_1041	Putative ABC-type multidrug transporters, ATP-binding/permease protein	-3.1381	-2.9258
Ligands transportation across membrane	Gallo_1042	Putative ABC-type multidrug transporters, ATP-binding/permease protein	-2.9779	-2.5898
Ligands transportation across membrane	Gallo_1172	Putative ABC transporters, permease protein	-4.1248	-2.1596
Ligands transportation across membrane	Gallo_1224	Putative spermidine/putrescine ABC transporters, spermidine/putrescine-binding protein	-3.7523	-2.9422
Ligands transportation across membrane	Gallo_1225	Putative spermidine/putrescine ABC transporters, permease protein	-2.6229	-2.4697
Ligands transportation across membrane	Gallo_1226	Putative spermidine/putrescine ABC transporters, permease protein	-2.2420	-2.3960
Ligands transportation across membrane	Gallo_1282	Putative proline/glycine betaine ABC transporters, permease protein	-3.8579	-2.2637
Ligands transportation across membrane	Gallo_1283	Putative proline/glycine betaine ABC transporters, ATP-binding protein	-4.4506	-2.0119

Ligands transportation across membrane	Gallo_1284	Putative proline/glycine betaine ABC transporters, substrate-binding protein	-5.0981	-2.4639
Ligands transportation across membrane	Gallo_1299	Putative ABC-type multidrug transporters, ATP-binding/permease protein	-2.5724	-2.6940
Ligands transportation across membrane	Gallo_1300	Putative ABC-type multidrug transporters, ATP-binding/permease protein	-2.6572	-2.0567
Ligands transportation across membrane	Gallo_1919	Putative sulfonate ABC transporters, ATP-binding protein	-2.5969	-4.0894
Ligands transportation across membrane	Gallo_1920	Putative sulfonate ABC transporters, permease protein	-2.5035	-4.1822
Ligands transportation across membrane	Gallo_1921	Putative sulfonate ABC transporters, substrate-binding protein	-2.0983	-3.3076
Pilus 3 biosynthesis	Gallo_2038	SrtC	-5.2687	-4.7045
Pilus 3 biosynthesis	Gallo_2039	Pil3B	-3.6501	-3.5732
Pilus 3 biosynthesis	Gallo_2040	Pil3A	-4.7754	-2.7881
Transcription	Gallo_0210	Transcriptional regulator, MarR family	-2.4851	-3.9567
Transcription	Gallo_1212	Putative transcription anti terminator BglG family	-2.1111	-2.0056
Transcription	Gallo_1286	Putative transcriptional regulator, MarR family	-3.9529	-2.2224
DNA replication, recombination and repair	Gallo_0978	ParC; DNA topoisomerase IV subunit A	-2.3698	-2.6929
DNA replication, recombination and repair	Gallo_0007	Mfd; transcription repair coupling factor	-2.7378	-3.6222
DNA replication, recombination and repair	Gallo_1925	Putative A/G-specific adenine glycosylase	-3.3180	-2.0135
Nucleotide transport and metabolism	Gallo_0704	NrdF; Ribonucleoside-diphosphate reductase (minor subunit)	-10.0501	-2.3125
Nucleotide transport and metabolism	Gallo_1449	Putative hydrolase	-2.6656	-2.0092
Nucleotide transport and metabolism	Gallo_1474	Putative 5'-Methylthioadenosine/S-adenosylhomocysteine nucleosidase	-3.8207	-2.0309
Translation, ribosomal structure and biogenesis	Gallo_0006	Pth; Peptidyl-tRNA hydrolase	-3.1115	-3.9031
Translation, ribosomal structure and biogenesis	Gallo_0373	RbfA; ribosome binding factor A	-4.5914	-2.4593
Carbohydrate transport and metabolism	Gallo_0183	Putative PTS system, mannitol/fructose-specific, IIC component	-2.0946	-2.6001
Amino acid transport and metabolism	Gallo_1468	HisB; Imidazoleglycerol-phosphate dehydratase	-2.0256	-2.8221
Amino acid transport and metabolism	Gallo_1469	SerB; phosphoserine phosphatase	-2.0495	-2.5757
Coenzyme transport and metabolism	Gallo_1230	FolB; Putative dihydroneopterin aldolase	-2.2070	-2.3828
Coenzyme transport and metabolism	Gallo_0211	Putative NADPH-dependent FMN reductase	-2.5166	-2.7520
Coenzyme transport and metabolism	Gallo_1160	RibF; Putative riboflavin kinase/FAD synthetase	-2.3192	-2.0414
Energy production and conversion	Gallo_0870	Putative Pyruvate/2-oxoglutarate dehydrogenase complex, dihydrolipoamide dehydrogenase (E3) component	-3.5310	-2.3151
Cell wall/membrane/envelope biogenesis	Gallo_1371	Putative glycosyltransferase	-2.5386	-3.6903
Unknown	Gallo_0703	Conserved hypothetical membrane protein	-3.4322	-2.4818
Unknown	Gallo_0720	Conserved hypothetical protein	-5.4100	-2.1619
Unknown	Gallo_0739	Conserved hypothetical protein	-2.0009	-3.0087
Unknown	Gallo_0815	Conserved hypothetical protein	-2.7158	-6.5851
Unknown	Gallo_1039	Conserved hypothetical protein	-2.7108	-2.5887
Unknown	Gallo_1091	Conserved hypothetical protein	-2.9080	-3.1903
Unknown	Gallo_1369	Conserved hypothetical protein	-2.0555	-3.0494
Unknown	Gallo_1408	Conserved hypothetical protein	-3.2086	-2.9453
Unknown	Gallo_1926	Conserved hypothetical protein	-2.5550	-2.1053
Unknown	Gallo_2206	Conserved hypothetical protein	-2.0666	-2.5841
Unknown	Gallo_2229	Conserved hypothetical membrane protein	-9.6665	-2.2782

Supplemental Data S2. Genes differentially expressed in *S. gallolyticus* UCN34 ∆*gdpP* mutant. 109 genes that were differentially expressed in the *S. gallolyticus* UCN34 ∆*gdpP* mutant, compared to the wild type and the *gdpP*-complemented strain. Raw sequencing reads were processed with CLC Genomics Workbench 8.2 and further analyzed using DESeq2 package. Numbers in column 4 and 5 indicate the fold change of the gene expression in ∆*gdpP*, compared to the wild type, or the *gdpP*-complemented strain, respectively.
